# Vaccines against Meningococcal Diseases

**DOI:** 10.3390/microorganisms8101521

**Published:** 2020-10-03

**Authors:** Mariagrazia Pizza, Rafik Bekkat-Berkani, Rino Rappuoli

**Affiliations:** 1 GSK, 53100 Siena, Italy; mariagrazia.x.pizza@gsk.com; 2 GSK, Rockville, MD 20850, USA; rafik.x.bekkat-berkani@gsk.com

**Keywords:** *Neisseria meningitidis*, meningococcal infection, invasive meningococcal disease (IMD), meningococcal outbreaks, meningococcal vaccines, reverse vaccinology, 4CMenB, rLP2086, outer member vesicles (OMV), bactericidal activity

## Abstract

*Neisseria meningitidis* is the main cause of meningitis and sepsis, potentially life-threatening conditions. Thanks to advancements in vaccine development, vaccines are now available for five out of six meningococcal disease-causing serogroups (A, B, C, W, and Y). Vaccination programs with monovalent meningococcal serogroup C (MenC) conjugate vaccines in Europe have successfully decreased MenC disease and carriage. The use of a monovalent MenA conjugate vaccine in the African meningitis belt has led to a near elimination of MenA disease. Due to the emergence of non-vaccine serogroups, recommendations have gradually shifted, in many countries, from monovalent conjugate vaccines to quadrivalent MenACWY conjugate vaccines to provide broader protection. Recent real-world effectiveness of broad-coverage, protein-based MenB vaccines has been reassuring. Vaccines are also used to control meningococcal outbreaks. Despite major improvements, meningococcal disease remains a global public health concern. Further research into changing epidemiology is needed. Ongoing efforts are being made to develop next-generation, pentavalent vaccines including a MenACWYX conjugate vaccine and a MenACWY conjugate vaccine combined with MenB, which are expected to contribute to the global control of meningitis.

## 1. Introduction

Invasive meningococcal disease (IMD) is a life-threatening disease caused by the bacterium *Neisseria meningitidis*. The most prominent presentations of IMD are meningitis and sepsis but other manifestations are also possible [1]. The onset of IMD is very sudden and the disease can be fatal within 24 to 48 h after developing symptoms, with high case fatality ratios of up to 20% [2,3]. Those who survive the disease often suffer from long-term debilitating sequelae, such as hearing loss, neurodevelopmental disease, and amputations [1]. 

The meningococcus is categorized into 12 meningococcal serogroups based on the bacterial capsular polysaccharide. Serogroups A, B, C, W, X, and Y most commonly cause IMD [4]. Meningococcal disease incidence varies geographically and over time [4,5,6]. Incidence is highest in infants, often followed by a second peak in adolescents and young adults [6]. 

*N. meningitidis* only infects humans and transmission occurs via direct contact with respiratory droplets from an infected person. Nasopharyngeal colonization occurs in up to 10% of the general population [3]. While carriage in the nasopharynx is typically asymptomatic, it can evolve into disease when bacteria enter the blood stream [6]. Carriage is highest in adolescents and young adults, mostly due to their lifestyle involving smoking, kissing, visits to bars, pubs and nightclubs, and living in close quarters [3,7,8]. Carriage rates are generally lower in older adults and infants [7].

IMD may occur sporadically, in small clusters, or evolve into large epidemics or outbreaks throughout the world [3].

Vaccination is regarded as the best strategy for the prevention of IMD due to the rapid onset and quick progression of the disease, and it can lower IMD-associated costs [9,10]. Great progress has been made in the control and prevention of IMD through the development and use of meningococcal vaccines [11]. The first vaccines based purely on capsular polysaccharides against serogroups A, C, W, and Y were advantageous but were not very effective in infants, had a short duration of protection, and could not induce immune memory. The development of meningococcal polysaccharide-conjugate vaccines to serogroups A, C, W, and Y (MenACWY), by conjugating the polysaccharide antigen to a carrier protein, overcame these limitations and made it possible to help protect young children who are at the highest risk for IMD. Additionally, meningococcal conjugate vaccines made it possible to reduce or prevent nasopharyngeal carriage, and thereby had the ability to induce herd protection [11,12]. 

Different MenACWY formulations have been available for a while and their use is recommended or offered as part of national immunization programs (NIPs) in many countries worldwide [13]. The development of a serogroup B (MenB) vaccine has been more challenging, but two broadly protective protein-based MenB vaccines are now also available [14].

**Figure 1:**
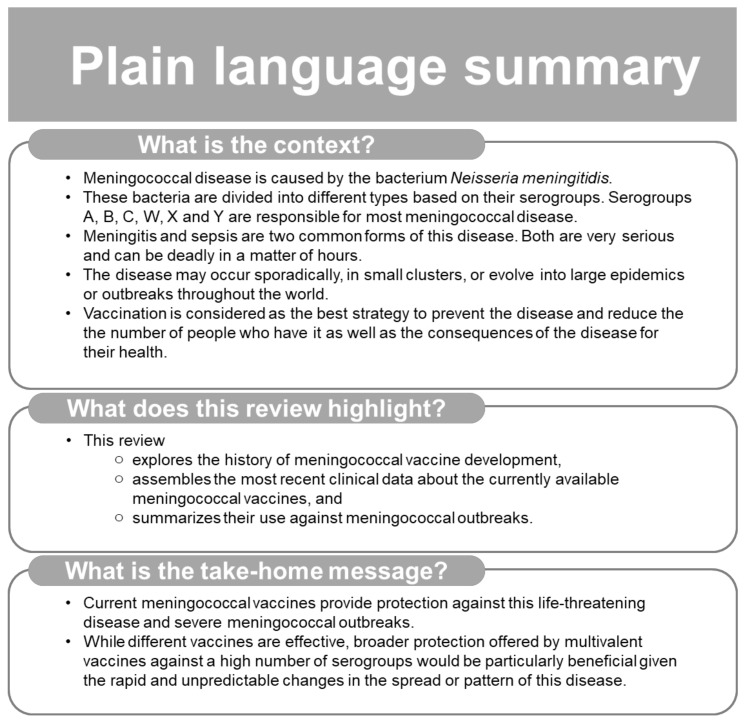
Plain language summary.

This narrative review explores the history of meningococcal vaccine development and assembles the most recent clinical data about the currently available meningococcal polysaccharide-conjugate vaccines and protein-based meningococcal vaccines. It also summarizes the use of meningococcal vaccines in meningococcal disease outbreaks and briefly discusses future challenges with regards to meningococcal vaccination. Figure 1 displays a plain language summary of this article for the reader.

**Figure 2:**
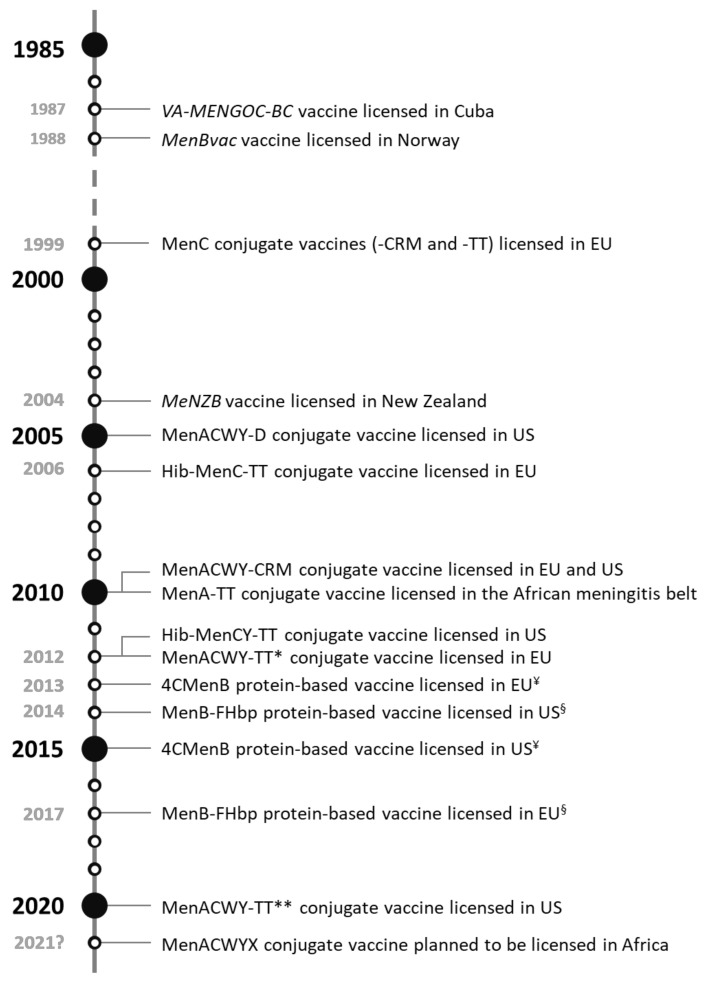
Timeline of the licensure of meningococcal conjugate, OMV-based and protein-based vaccines. *VA-MENGOC-BC* (Finlay Institute), OMV-based vaccine licensed in Cuba; *MenBvac* (Norwegian Institute of Public Health), OMV-based vaccine licensed in Norway; MenC-CRM (*Meningitec*, Nuron Biotech and *Menjugate*, GSK), meningococcal serogroup C conjugate vaccine conjugated to diphtheria protein cross-reactive material 197; MenC-TT (*NeisVac-C*, Pfizer), meningococcal serogroup C conjugate vaccine conjugated to tetanus toxoid; CRM, diphtheria protein cross-reactive material 197; TT, tetanus toxoid; EU, European Union; *MeNZB* (Novartis), OMV-based vaccine licensed in New Zealand; MenACWY-D (*Menactra,* Sanofi Pasteur), quadrivalent meningococcal conjugate vaccine conjugated to diphtheria toxoid; USA, United States; Hib-MenC-TT (*Menitorix,* GSK), *Haemophilus influenzae* type b-*Neisseria meningitidis* serogroup C-tetanus-toxoid conjugate vaccine; MenACWY-CRM (*Menveo,* GSK), quadrivalent meningococcal conjugate vaccine conjugated to diphtheria protein cross-reactive material 197; MenA-TT (PsA-TT, *MenAfriVac*, Serum Institute of India), monovalent meningococcal serogroup A conjugate vaccine conjugated to tetanus toxoid; Hib-MenCY-TT (*MenHibrix*, GSK), *Haemophilus influenzae* type b-*Neisseria meningitidis* serogroups C and Y-tetanus-toxoid conjugate vaccine; MenACWY-TT* (*Nimenrix*, Pfizer), quadrivalent meningococcal conjugate vaccine conjugated to tetanus toxoid; 4CMenB (*Bexsero,* GSK), 4-component meningococcal serogroup B protein-based vaccine; MenB-FHbp (*Trumenba*, Pfizer), bivalent meningococcal serogroup B protein-based vaccine; MenACWY-TT** (*MenQuadfi*, Sanofi Pasteur), quadrivalent meningococcal conjugate vaccine conjugated to tetanus toxoid; MenACWYX (*NmCV-5*, Serum Institute of India), pentavalent meningococcal conjugate vaccine. ^¥^ 4CMenB is licensed for use in infants from 2 months of age in the EU and in 10–25-year-olds in the USA; ^§^ MenB-FHbp is licensed for use in individuals aged 10 years and older in the EU and in 10–25-year-olds in the USA.

Figure 2 shows a timeline of meningococcal conjugate, outer member vesicles (OMV)-based and protein-based vaccine licensure.

## 2. Meningococcal Polysaccharide-Conjugate Vaccines

### 2.1. Monovalent Conjugate Vaccines

#### 2.1.1. Meningococcal Serogroup C Conjugate Vaccines

The United Kingdom (UK) was the first country to introduce MenC conjugate vaccination, in 1999, after experiencing increases in MenC disease due to the sequence type (ST) 11 clonal complex, as also seen in multiple other countries. It was first introduced in the routine infant vaccination schedule at 2, 3, and 4 months of age and as a single-dose catch-up vaccination in 1–18-year-olds. Several other countries subsequently introduced MenC conjugate vaccines in their NIP. These programs have been highly successful in reducing MenC disease [15].

Three MenC conjugate vaccines have been licensed for use in the UK and other countries worldwide, i.e., two MenC vaccines conjugated to diphtheria protein cross-reactive material 197 (MenC-CRM) (*Meningitec*; Nuron Biotech and *Menjugate*, GSK), and one MenC vaccine conjugated to tetanus toxoid (MenC-TT) (*NeisVac-C*, Pfizer) [15].

These vaccines were licensed on the basis of immunogenicity studies, because sufficiently powered efficacy trials were considered to be unfeasible due to the rarity of the disease [16]. The serum bactericidal antibody (SBA) assay was established as a serologic correlate of protection for meningococcal disease [17]. The assay is based on complement-mediated killing of bacteria by serum antibodies. SBA titres greater than or equal to four with human complement (hSBA) and greater than or equal to eight with baby rabbit complement (rSBA) are indicative of protective efficacy [18]. High circulating levels of protective antibodies are important because the onset of disease is so rapid that the production of antibodies in response to infection is too slow to be protective [15,19].

Clinical trials of all MenC conjugate vaccines demonstrated that they were immunogenic and had an acceptable safety profile in all age groups, and were able to induce a robust booster response through immune memory up to four years after completion of infant immunization [19]. There are small differences among the MenC conjugate vaccines in immunogenicity and avidity, possibly due to the different carrier protein, conjugation chemistry, acetylation of the MenC polysaccharide, or length of polysaccharide constituents. *NeisVac-C* showed greater immunogenicity and antibody persistence than *Meningitec* or *Menjugate* [15]. 

All licensed MenC conjugate vaccines demonstrated good immune responses in adolescents. Waning of protection is seen in all age groups (infants, toddlers, and adolescents) with all MenC conjugate vaccines [15]. 

Many countries have implemented MenC conjugate vaccines in their NIP using different schedules and targeting different age groups. Effectiveness of approximately 90% in preventing MenC disease has been demonstrated for all MenC vaccines regardless of the conjugation protein, underscoring the success of routine vaccination programs [15]. 

The national MenC vaccination program in the UK had good coverage of above 90% in infants, above 75% in preschool children and above 85% of school children aged 5 to 16 years old. After implementation, dramatic reductions in MenC IMD were observed in vaccine-targeted age groups demonstrating high effectiveness [15]. The lowest recorded incidence was 0.02 per 100,000 population in epidemiological years 2008/2009 [15] and low levels were maintained with an incidence of 0.08/100,000 in 2015/2016 [20]. Importantly, the impact of MenC disease was also observed in unvaccinated individuals as a result of herd protection from the catch-up program [15]. 

Herd protection has also been observed in the Netherlands, Canada, and Australia following MenC conjugate vaccination [15]. A nationwide study in the Netherlands showed reductions in MenC disease of 99% in vaccinated age groups and 93% in unvaccinated age groups a decade after routine use of MenC conjugate vaccine at 14 months and catch-up campaigns in those between 1 and 18 years of age, demonstrating evidence for herd protection [21]. In Canada, implementation of a mass vaccination campaign in the population aged 2 months to 20 years (followed by routine use at 12 months) dramatically reduced MenC disease incidence in vaccinated and non-vaccinated individuals [22,23]. In Australia, ten years after introduction of MenC conjugate vaccine in the NIP at 12 months of age, MenC disease had decreased by 96%, and herd effects were observed in unvaccinated age groups [24]. In France, the introduction of MenC conjugate vaccination in the NIP in 2010 had a more limited impact on reducing MenC disease incidence. In age groups not targeted by the vaccine, annual incidence rates increased between 2010 and 2015. This can be explained, mainly, by the emergence of a new MenC epidemic cycle in 2011 and low vaccine coverage among adolescents and young adults at that time. This highlights the importance of maintaining high vaccine coverage, especially in adolescents and young adults, in order to sustain long-term direct and indirect protection from disease [25].

Over time, the UK immunization schedule has evolved based on the learnings from its widespread use and implementation, the development of new vaccines, and changing disease epidemiology [20]. Many countries (for example the UK and Canada) have now introduced booster vaccinations in adolescents to maintain high levels of antibodies in this age group, likely reducing carriage, and thus maintaining herd protection in older children and adults, which is considered as a major player in the success of the MenC vaccination program [26].

In addition to monovalent MenC conjugate vaccines, there are combination conjugate vaccines that include *Haemophilus influenzae* type b (Hib). Hib-MenC-TT (*Menitorix*, GSK) has been licensed since 2006 and is mostly used as a booster dose in toddlers in the UK and Australia and Hib-MenCY-TT (*MenHibrix*, GSK) was licensed in the United States (USA) as of 2012 but has been discontinued [11,27,28].

In the UK, emergency vaccination with MenACWY (MenACWY-CRM or MenACWY-TT) was introduced for 13–18-year-olds in 2015/2016 in response to a MenW outbreak [29]. Increasingly, other countries have also transitioned to quadrivalent conjugate vaccines for broader protection against disease-causing strains (as further discussed below). 

#### 2.1.2. Meningococcal Serogroup A Conjugate Vaccines

Historically, the highest incidence of IMD in the world occurs in countries in the African meningitis belt, a region in sub-Saharan Africa stretching from Senegal to Ethiopia. During epidemics, mainly due to serogroup A, but also due to other serogroups, incidence rates can reach as high as 1% of the population [5]. The disease has a seasonal pattern and occurs mostly during the dry season from December to June [30]. The meningococcal serogroup A polysaccharide-tetanus toxoid conjugate vaccine (PsA-TT (MenA-TT), *MenAfriVac*, Serum Institute of India) was specifically designed to control MenA disease spread in the region [31]. This vaccine does not require a cold chain and can be used at ambient temperature for up to four days [32]. It is available in two formulations, with either 5 µg or 10 µg of purified MenA polysaccharide antigen conjugated to TT, for immunizing infants and children aged 3 to 24 months and persons aged 1 to 29 years old, respectively [31].

PsA-TT was introduced in the African meningitis belt in different phases, starting in late 2010, in mass immunization campaigns targeting individuals aged 1 to 29 years old. Data from the enhanced meningitis surveillance network supported by the World Health Organization (WHO) showed that PsA-TT introduction led to dramatic decreases in the incidence levels of suspected meningitis (57%), epidemic risk (59%), and confirmed serogroup A disease (more than 99% in fully vaccinated populations) in nine countries through 2015 [33]. 

Two studies in Ghana and Mali in infants and young children demonstrated the immunogenicity and safety of the vaccine when co-administered with other routine vaccines and also the ability to induce immune memory [31].

Several countries in the meningitis belt are now implementing the vaccine into their routine Expanded Programme on Immunization schedule targeting age 9–18 months [30,33], as recommended by the WHO. The WHO recommends introduction into routine childhood immunization at 9 months of age, within one to five years after completing the initial mass vaccination campaigns, with catch-up campaigns targeting unvaccinated children aged one to four years [31]. 

The need for a booster dose has not been completely established. The long-term effectiveness of PsA-TT was investigated in two clinical trials that followed antibody persistence up to five years post-vaccination and found that after an initial decline of antibody titres during the first year post-vaccination, antibody titres remained stable in all age group between 1 and 29 years [34]. In addition, extrapolation of population level data from a series of cross-sectional studies in Burkina Faso estimated that SBA titres would return to pre-vaccination levels after at least 12 years. A mathematical model from White et al. [35] also predicted a vaccine efficacy of 52% at 20 years after a single dose in children 12 to 23 months of age and 70% in older children and adults, suggesting that no booster vaccination would be needed. However, there is no confirmed correlate of protection against MenA in the meningitis belt [34,35].

Vaccination with PsA-TT has been reported to reduce carriage of MenA. In Burkina Faso, a reduction from 0.39% baseline prevalence to no MenA carriage in oropharyngeal samples was observed over a period from three weeks up to 13 months after mass vaccination from vaccinated as well as unvaccinated populations [36]. In Chad, reductions of up to 98% in carriage of MenA were observed after vaccine introduction [37]. A large longitudinal carriage study from the African Meningococcal Carriage Consortium (MenAfriCar) was conducted across seven countries of the meningitis belt [38]. MenAfriCar reported via cross-sectional, standardized surveys that carriage was infrequent and dynamic in Africa, even in the absence of vaccination [39]. 

In addition to the above vaccines, regional conjugate vaccines against combined MenAC are also available and used in China [11].

### 2.2. Multivalent Conjugate Vaccines

Epidemiology of IMD has changed after the introduction of meningococcal vaccines [6], as exemplified by the emergence of MenW disease in several countries across the world [40,41]. This has resulted in changes in vaccine recommendations from MenC to MenACWY vaccines in the UK and other countries [42].

**Figure 3:**
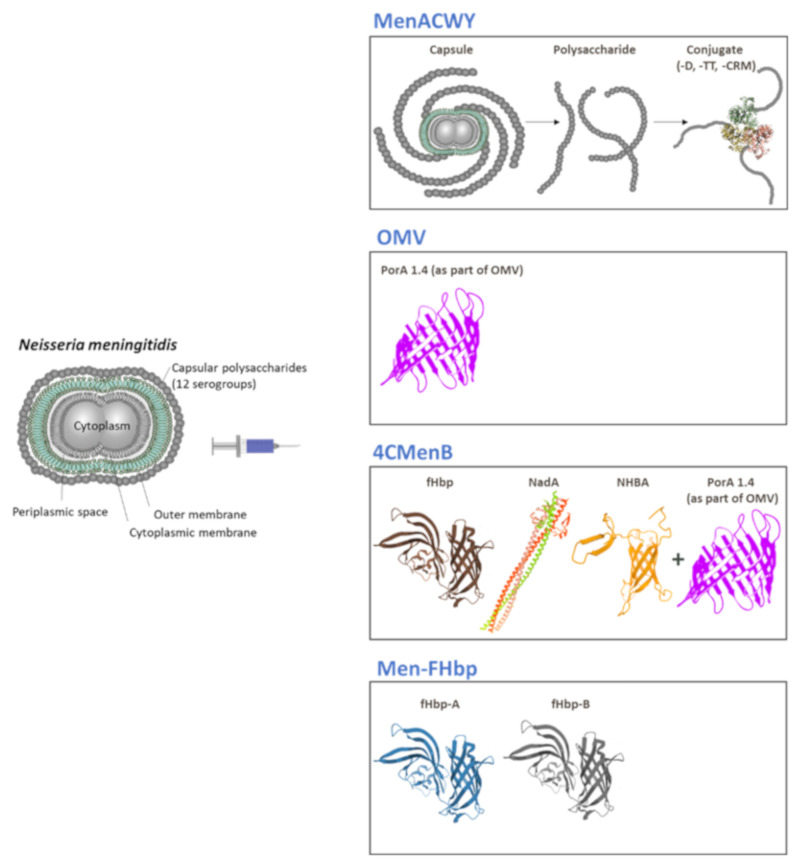
Structure of the meningococcus and overview of the different antigens used in meningococcal conjugate and protein-based vaccines. Abbreviations: D, diphtheria toxoid; TT, tetanus toxoid; CRM, diphtheria protein cross-reactive material 197; PorA 1.4, Porin A protein; OMV, outer membrane vesicles from New Zealand strain NZ98/254 containing PorA 1.4; fHbp, factor H binding protein; NadA, Neisseria adhesin A; NHBA, Neisserial heparin binding antigen; fHbp-A and -B, variants of fHbp from 2 subfamilies. Antigen images from the RCSB PDB (rcsb.org) of PDB ID 3KVD [44], 6EUN [45] and 2LFU [46], Mol* [47]. The PorA image reproduced with permission from Derrick et al. [48].

There are currently four formulations of quadrivalent meningococcal conjugate vaccines available globally. All contain capsular polysaccharides of MenA, MenC, MenW, and MenY but they differ in the type of carrier protein. MenACWY-D (*Menactra*, Sanofi Pasteur) is conjugated to diphtheria toxoid (D), MenACWY-CRM (*Menveo*, GSK) is conjugated to CRM, while two MenACWY-TT vaccines are conjugated to TT, *Nimenrix* (Pfizer) and the recently available *MenQuadfi* (Sanofi Pasteur) (Figure 3) [11,43].

MenACWY-D was the first quadrivalent meningococcal conjugate vaccine. It has been licensed since 2005 in the USA and is currently approved for infants 9–23 months old as two doses and for individuals from 2 to 55 years old as a single dose [49]. Currently it is not approved in Europe [50]. It can be co-administered with other vaccines, but the immunogenicity of some co-administered vaccines is affected [49]. Protective antibody levels have been described up to three years after vaccination of adolescents, but reduced MenACWY-D effectiveness five years after primary vaccination has been observed due to declining antibody levels [49].

Some of the more recent clinical data investigated the administration of a single booster dose in adolescents and young adults who received a previous dose four to six years earlier and showed robust booster responses indicative of immune memory with no safety concerns [51]. In a follow-up study, though antibody levels had declined substantially, more than 81% of participants retained levels of bactericidal antibodies (hSBA ≥ eight) four years after the booster dose [52].

Early effectiveness of MenACWY-D estimated in a U.S. study within three to four years after vaccination was approximately 80–85% against MenC and MenY disease in adolescents [53].

MenACWY-CRM was first licensed, in 2010, in the USA and also in the European Union (EU) [13,54]. It is currently approved in persons from two months to 55 years of age in the USA and other countries and from two years of age in the EU [13]. MenACWY-CRM is indicated for use as a three-dose series in healthy infants from two to six months of age, a four-dose series in infants from two to six months of age at high risk for IMD, a two-dose series between 7 and 23 months of age, and as a single dose from two years of age in the USA and other countries [55,56]. Several countries have integrated MenACWY-CRM in their NIP for use in infants [13], and some countries, including Argentina [57] and Switzerland [58], also for use in adolescents.

MenACWY-CRM has been shown to induce seroprotective antibody titres (hSBA greater than or equal to eight) in the majority of vaccinated infants, children, adolescents, and adults from two months to 75 years of age. It can be administered alone or with other routine vaccines in any age group without clinically meaningful interactions on the immune response to MenACWY-CRM or co-administered vaccines and has a favorable benefit-risk profile. Antibody persistence was demonstrated up to five years after vaccination in all age groups. Robust booster responses for all serogroups were induced after booster vaccination in all age groups, indicative of immune memory [13].

The superiority of two doses of MenACWY-CRM, as compared with a single dose, was demonstrated in two- to five-year-old children in the USA in terms of percentage of subjects with seroresponse against serogroups C and Y one month after last vaccination, whereas superiority of two doses was demonstrated in six- to ten-year-old children only for serogroup Y. In the same study, antibody persistence one year post-vaccination was demonstrated to be higher for serogroups A and C in two- to five-year-old children and serogroups C and Y in six- to ten-year-old children who received two doses versus one dose [59]. In another study comparing one versus two doses of MenACWY-CRM, geometric mean titres and proportions of children with hSBA titres ≥eight were significantly higher in two- to five-year-old children who received two doses of MenACWY-CRM than in those who received only one dose [60]. However, these differences no longer existed when assessing antibody persistence in both groups five years after vaccination [61].

Recent clinical results showed that a booster dose of MenACWY-CRM in adolescents and young adults, administered four to six years after priming, induced robust anamnestic responses, regardless of MenACWY-CRM or MenACWY-D priming [62]. 

Early research suggested a significant impact of MenACWY-CRM on carriage of MenY and combined serogroups CWY in university students from 2 to 12 months after a single dose [63]. Recently, a study in the Korean Armed Forces, a high transmission group, in the Republic of Korea showed a reduction of 88% in meningococcal disease incidence during a 19–23-month observation period after vaccinating more than 1.5 million soldiers with a single dose of MenACWY-CRM [64].

MenACWY-TT (*Nimenrix*, Pfizer) was approved in 2012, in Europe, in infants aged six weeks or greater with no upper age limit [50]. It is indicated for use as a two-dose series in healthy infants from six weeks to less than six months of age followed by a booster dose at 12 months of age, and as a single dose from six months of age [65]. It is currently not approved for use in the USA [50]. 

The immunogenicity, persistence, and safety profile of MenACWY-TT have been established in multiple clinical studies across various age groups (infants, toddlers, children, adolescents, and young adults) while co-administered with routine vaccines [50]. A booster dose in persons previously vaccinated with a conjugate or polysaccharide meningococcal vaccine has also been shown to be immunogenic and safe [66]. 

In adolescents and young adults who were primed at age 10–25 years with a single dose of MenACWY-TT or MenACWY-D, antibody persistence up to five years after priming has been reported, as well as robust responses to a MenACWY-TT booster dose given five years after priming. This is supportive of induction of immune memory after primary vaccination with MenACWY-TT. Antibody persistence up to five years after primary vaccination was also assessed in other studies. Additionally, a MenACWY-TT booster dose in adolescents who had been primed with a MenC vaccine, induced robust antibody responses [66].

MenACWY-TT has been shown to reduce carriage in a few smaller studies in university students in the UK and military personnel in Poland [8]. 

The second MenACWY-TT vaccine (*MenQuadfi*, Sanofi Pasteur) was approved by the Food and Drug Administration in April 2020 for use in persons two years of age and older in the USA. It was licensed based on non-inferiority of immune responses as compared with those elicited by the other U.S.-licensed quadrivalent meningococcal vaccines according to the age group [67]. In a recent exploratory phase 2 study, the immunogenicity and safety of *MenQuadfi* was reported to be comparable with the licensed MenACWY-TT in toddlers aged 12–24 months old, although responses to serogroups A and C differed between the vaccines [68]. Non-inferiority in terms of seroresponse rates at day 30 was demonstrated versus MenACWY-CRM in a phase 2 study in adolescents between 10 and 17 years of age [69]. In an exploratory phase 2 study in adults aged 56 years or older, the proportion of participants achieving hSBA titres ≥8 for serogroups A and C was comparable to those after a licensed quadrivalent meningococcal polysaccharide vaccine (*Menomune*; Sanofi Pasteur), but higher for serogroups W and Y after receiving *MenQuadfi*. Proportions of participants with rSBA titres greater than or equal to eight were comparable between vaccine groups for all four serogroups [70]. A booster dose was also evaluated in a phase 3 study in adolescents and adults and induced robust immune responses four to ten years after priming with MenACWY-D or MenACWY-CRM [71]. The Advisory Committee on Immunization Practices has recently approved *MenQuadfi* as an alternatively recommended MenACWY vaccine in the USA [72]. This vaccine is currently under regulatory review in Europe [67].

A low-cost pentavalent meningococcal conjugate vaccine (MenACWYX, *NmCV-5*, Serum Institute of India) is in development for Africa and planned to be implemented in 2020 or 2021. This vaccine contains serogroup A and X polysaccharides individually conjugated to TT and serogroup C, W, and Y polysaccharides individually conjugated to CRM [43].

## 3. Meningococcal Protein-Based Vaccines

Unlike the above vaccines, no polysaccharide-conjugate vaccines exist for serogroup B. The polysaccharide of MenB is poorly immunogenic and can potentially cause autoimmune reactions because its chemical composition is identical to polysialic acid found on the surface of many human cells [14]. The development of a MenB vaccine has been a major challenge. The two vaccines currently available to prevent MenB disease, 4CMenB (*Bexsero*, GSK) and MenB-FHbp (*Trumenba*, bivalent rLP2086, Pfizer), were each developed using a different strategy but are both protein-based vaccines. These vaccines were urgently needed as MenB became responsible for the majority of disease in countries with a meningococcal vaccination program [73].

### 3.1. Outer Membrane Vesicles (OMV)-Based Vaccines

Historically, MenB vaccines were based on purified OMV treated with detergents to extract lipooligosaccharide and to decrease endotoxin activity. Their immunodominant antigen was the meningococcal outer membrane protein porin A (PorA). These OMV-based vaccines were designed for protection against clonal outbreaks and were strain specific (especially in infants). They were protective only if the PorA serosubtype of a disease-causing outbreak clone matched that of the OMV vaccine [14]. 

Tailor-made OMV vaccines have shown their effectiveness to control epidemic outbreaks in Cuba, Norway, and New Zealand. In Cuba, a first OMV vaccine, *VA-MENGOC-BC* (Finlay Institute) was developed and used in children aged 10–14 years old between 1987 and 1989. In Norway, a second OMV vaccine, *MenBvac* (Norwegian Institute of Public Health)*,* was used from 1988 to 1991 in children aged 13–16 years old. These vaccines both induced good bactericidal antibody responses against homologous MenB strains in infants and older age groups, but two doses were not sufficient to induce long-term protection in infants. An outbreak of a heterologous MenB strain in New Zealand led to the development of the *MeNZB* (Novartis) vaccine which was used in the New Zealand NIP as a three-dose schedule between 2004 and 2008, first in individuals older than six months, and later expanded to infants older than six weeks. By June 2016, more than three million doses were administered in those under 20 years old. There were no safety concerns identified. An effectiveness of 77% was estimated by Poisson regression over 3.2 years after a three-dose primary series, and it was reduced to 68% when considering potential residual confounding. There was also evidence of some effectiveness against non-B disease [74]. The *MenBvac* vaccine from Norway was also used to control a MenB outbreak caused by a genetically close strain expressing the same PorA serosubtype in Normandy, France, from 2006 to 2012 [75,76]. All those less than 20 years old who lived in Normandy from 2006 to 2009 were eligible to receive the vaccine according to an age-appropriate schedule. The incidence of confirmed MenB cases decreased significantly from 31.6 per 100,000 in the time before vaccination to 5.9 per 100,000 after primary vaccination [75]. An evaluation of antibody persistence at one and four years after *MenBvac* booster vaccination showed that it was short lived and that (repeated) booster doses may be required. The strain coverage by *MenBvac* to other B and non-B strains (that at least partially match PorA of the vaccine strain) may be greater than expected [76].

### 3.2. 4CMenB Vaccine

For the development of 4CMenB, the approach to identify antigens was different from the traditional laboratory-based approach. Instead, potential antigens were identified by whole genome sequencing in a process called reverse vaccinology. From the 600 antigens identified in silico, 28 were surface-exposed antigens able to induce a bactericidal antibody response. The three most promising antigens were selected for inclusion in the 4CMenB vaccine based on the extent of their variation within the natural population of meningococcal strains and on their ability to induce bactericidal activity against genetically diverse strains. These antigens were the following: Neisserial heparin binding antigen (NHBA), human factor H binding protein (fHbp), and Neisseria adhesin A (NadA). When each of NHBA and fHbp were fused with additional candidate antigens, their bactericidal activity was increased. The final 4CMenB formulation contained the three main antigens in combination with OMV from the epidemic New Zealand outbreak (Figure 3) [14]. 

In contrast to the meningococcal quadrivalent conjugate vaccines, determination of the immunogenicity of the 4CMenB vaccine candidates was challenging due to the diversity of invasive disease MenB target antigens. Four reference strains were chosen to selectively measure functional bactericidal antibody responses to each individual vaccine antigen. The four antigen-specific strains were not matched to any of the other three antigens. These reference strains were genetically diverse but were not selected to be representative of the genetic diversity of circulating MenB strains [14]. 

Due to different levels of expression and peptide diversity in vaccine antigens across meningococcal strains, estimating the effectiveness of 4CMenB against MenB disease would mean performing SBA assays against a very large number of strains, which was considered not feasible due to the large volumes of sera and human complement needed [73]. Therefore, the Meningococcal Antigen Typing System (MATS) was developed to estimate the potential coverage of 4CMenB. It measures the 4CMenB antigens in large panels of circulating MenB isolates without the need for human sera [14]. The MATS relies on viable cultures of invasive isolates. Internationally-standardized MATS estimated high coverage rates by 4CMenB worldwide, ranging from 66% to 91% in 14 countries [14]. Genetic MATS (gMATS) combines antigen genotyping with MATS to predict 4CMenB strain coverage without the need for a cultivable isolate. Results from both MATS and gMATS across European and non-EU MenB strain panels were concordant but both underestimated the effectiveness of 4CMenB (as assessed by killing in hSBA) because these systems do not consider cooperative effects between the antigens, or underestimated the contribution of the NadA antigen or minor OMV components of 4CMenB [77].

4CMenB was licensed in 2013, in Europe, for use in infants from two months of age as a three + one schedule based on immunogenicity and safety clinical trials alone [14,73].

The safety and immunogenicity of 4CMenB has been demonstrated in infants and children, as well as in adolescents and adults. A three-dose primary vaccination schedule and booster dose were demonstrated to be immunogenic in infants, while a two-dose vaccination schedule was immunogenic in toddlers and children between 2 and 10 years of age [73]. 4CMenB can be co-administered with most of the routinely recommended childhood vaccines. While antibodies waned after priming in infants and children, persistence up to 36 months after various schedules of 4CMenB and robust booster responses indicative of immune memory were reported [73,78]. Studies performed in adolescents and adults established the immunogenicity of 4CMenB after two doses [73]. Recent clinical data demonstrated antibody persistence and robust booster responses in adolescent and young adults at 4 and 7.5 years after primary vaccination [79].

Local and systemic reactions after 4CMenB administration are mostly transient and mild-to-moderate in nature. As fever occurs more frequently after 4CMenB co-administration with other routine vaccines in infants, prophylactic paracetamol has been recommended in the UK and Australia to reduce fever without influencing the immunogenicity of the vaccines. In adolescents, pain at the injection site has been the most commonly reported reaction, while fever has been uncommon [73]. 

The effects of 4CMenB on carriage have been studied in one phase 3 randomized clinical trial. This study showed that MenACWY-CRM and 4CMenB reduced the carriage of *N. meningitidis* by 36% and 27%, respectively, during the year after vaccination [63,73]. By contrast, no differences in carriage were observed 12 months after vaccination in the 4CMenB or the control group in a cluster-randomized study in South Australia from April to June 2017, in which 34,489 students aged 15 to 18 years participated [80]. More studies are needed to better understand the effects of 4CMenB on carriage.

4CMenB was first introduced into the NIP in the UK, in 2015, using a reduced two + one (2, 4, and 12 months) schedule in infants [14]. After the UK, a number of other countries have now implemented 4CMenB in their NIP, including Andorra [81], Ireland [82], Italy [83], and Lithuania [84], whereas in the USA the vaccine received a Category B national recommendation [85].

Early vaccine effectiveness in the UK against all MenB IMD cases after two doses was 83% (95% confidence interval (CI) 24–95) [86]. After the first three years of the NIP in the UK, significant reductions in the incidence of MenB disease (75%) were observed in vaccine-eligible cohorts. The adjusted vaccine effectiveness over the course of three years using an indirect screening method was 53% (95% CI -34–83) after two doses and 59% (95% CI -31–87) after three doses [87]. 

In Quebec, Canada, following a local outbreak of MenB disease in the Saguenay-Lac-Saint-Jean region, a 4CMenB mass immunization program of individuals from two months up to 20 years was implemented in 2014. No IMD cases were reported in vaccinated persons, while two IMD cases in unvaccinated adults and one IMD case in an unvaccinated child were reported two years after the campaign start [88]. In a follow-up of this study four years after the campaign, vaccine effectiveness was estimated at 79%, with most of the campaign effects in the target age group, and therefore not suggestive of a herd effect in unvaccinated persons [89].

Further evidence demonstrating the effectiveness of 4CMenB is expected to come from South Australia where 4CMenB has been implemented in the NIP as of October 2018, to vaccinate those at highest risk, including infants (six weeks to 12 months of age), young children (one to three years of age), and adolescents and young adults (15 to 20 years of age) [90]. Additional evidence of effectiveness is expected to come from Italy and Portugal.

4CMenB is also approved in the USA, since 2015, for use in individuals aged 10–25 years old as a two-dose schedule. It has been used there to control outbreaks at universities [91,92,93,94]. 

### 3.3. MenB-FHbp Vaccine

The MenB-FHbp vaccine contains two lipidated recombinant fHbp variants from two subfamilies (Figure 3) [95]. The identification of fHbp as a target for a MenB vaccine occurred via a biochemical approach. MenB-FHbp has been approved in Europe (since 2017) and Australia for individuals of 10 years of age or older. It has also been approved in the USA, since 2014, as well as in Canada, for persons aged 10 to 25 years of age [96,97]. A two-dose series (administered at zero and six months) and a three-dose series (administered at zero, one to two, and six months) have both been approved for use [97].

The vaccine development program of MenB-FHbp focused on immunogenicity, persistence, and safety in adolescents and young adults given the important role of these age groups in disease transmission [96]. MenB-FHbp can be safely co-administered with other recommended adolescent vaccines without immunological interference and has been shown to induce immune responses against antigenically and epidemiologically diverse MenB isolates. With respect to 4CMenB, the selection of MenB test strains was critical for vaccine assessments. Four representative test strains were initially chosen out of 1263 MenB isolates, two for fHbp variant A and two for variant B, to assess immune responses to the vaccine by hSBA. The initial test strains were heterologous for the vaccine antigens and represented common multilocus sequence types [96], but were not representative of the overall fHbp expression levels among disease-causing MenB isolates, which could vary significantly among strains [98]. In later phase 3 studies, ten additional MenB test strains were selected to evaluate the broad coverage of the vaccine against the variety of MenB disease-causing strains. Immunogenicity assessments used for licensure included a combined response to all four test strains, defined as hSBA titres of at least the lower limit of quantitation, i.e., 1:8 or 1:16 depending on the test strain. The 120 µg dose was selected for further clinical development based on early phase 1 and 2 studies [96]. 

Similar to MATS which is used to predict 4CMenB coverage, predictions of coverage by MenB-FHbp are estimated using a novel assay. The flow cytometric meningococcal antigen surface expression (MEASURE) assay quantifies only fHbp cell-surface expression levels on MenB isolates, independent of their antigenic diversity [95].

Two- or three-dose schedules of MenB-FHbp were immunogenic and produced similar immune responses after vaccination [96,97]. A two-dose schedule (zero and six months) might be more appropriate for routine vaccination, whereas the three-dose schedule (zero, one to two, and six months) might be more appropriate for persons at high risk for MenB disease or in response to MenB outbreaks [97]. 

Antibody persistence of the immune responses elicited by MenB-FHbp has been demonstrated in several clinical studies [97]. Recently, antibody persistence four years after a two- or three-dose MenB-FHbp primary vaccination schedule in healthy adolescents was evaluated in a phase 3 extension study. This study also assessed the safety and immunogenicity of a booster dose of MenB-FHbp administered four years after primary vaccination. While antibody levels declined initially up to 12 months after two- or three-dose vaccination, they stabilized from 12 to 48 months. Geometric mean titres measured one month after booster vaccination were comparable to or higher than those measured one month after the last primary vaccination dose, and were similar after two- or three-dose primary vaccination [99]. 

The clinical development of MenB-FHbp in infants was suspended following early termination of a phase 1/2 study due to high rates of fever after receiving a partial dose of the vaccine [73,95]. Studies evaluating the immunogenicity and safety of MenB-FHbp in toddlers and young children are currently ongoing or recently completed [95].

MenB-FHbp is not currently included in any NIP but received a Category B national recommendation in the USA, where it has been used to control university outbreaks [85,94]. Real-world evidence on the use of MenB-FHbp is limited. 

4CMenB and MenB-FHbp vaccines are not interchangeable due to their different antigen compositions and vaccination schedules [95].

Because both MenB vaccines are broad-coverage vaccines and contain vaccine antigens that are also present in other non-MenB strains, MenB vaccines can potentially provide cross-protection against disease caused by other serogroups. Serum samples from infants vaccinated with 4CMenB had potent SBA activity against the hypervirulent MenW strains circulating in the UK [100]. SBA data using pooled sera from infants, adolescents, and adults who received 4CMenB suggested that 4CMenB could provide coverage of nine African MenX invasive isolates but not of two unrelated MenX isolates from France [101]. There is also evidence that MenB-FHbp can induce protective bactericidal antibody responses against MenC, MenY, MenW, and MenX strains [95]. In addition, coverage of *Neisseria gonorrhoeae* by the OMV-based *MeNZB* vaccine [102] and by 4CMenB in Canada has been reported [103,104].

Investigation of a next-generation, pentavalent combination MenABCWY-CRM-conjugated vaccine has been started in adolescents and adults [105,106,107,108,109,110]. This vaccine combines MenACWY-CRM and the 4CMenB vaccine and has been developed to provide broader protection against the five most epidemiologically relevant serogroups.

## 4. Outbreaks

Outbreaks of meningococcal disease occur when the same serogroup causes multiple cases in a defined population over a short period of time [111]. In the African meningitis belt, where frequent epidemics occur, outbreaks are defined by the WHO as the occurrence of >100 cases/100,000 population/year [112].

A systematic review on meningococcal disease outbreaks in non-African countries from January 1966 until July 2017 identified MenC as the predominant serogroup in the 83 included outbreaks (61%), followed by MenB (29%), MenA (5%), and MenW (4%). During the 10-year period from 2006 until July 2017, the number of MenB outbreaks in the Americas increased, and MenW outbreaks emerged in the Americas and Europe [113]. As mentioned above, both 4CMenB and MenB-FHbp have been used to control university outbreaks in the USA [94]. The first large MenW outbreak started in 2000 among Hajj pilgrims in Saudi Arabia. Since 2002, vaccination with a MenW-containing vaccine has been mandatory for pilgrims travelling to Saudi Arabia [40]. 

The above systematic review reported a low number of outbreaks in South East Asia, the Western Pacific, and the Eastern Mediterranean region (<10 in each region over the 50-year period). Outbreaks mostly followed a seasonal pattern, with a peak in the colder months [113].

Prior to the introduction of PsA-TT, outbreaks in the African meningitis belt were mainly due to MenA. Following the use of PsA-TT and its ability to provide protection against MenA disease, outbreaks due to MenC, MenX, and MenW are now the public health concern in these countries. The largest MenC outbreak to date took place in Nigeria in 2013 and Niger in 2015, due to a previously unrecorded MenC clone [30]. While the largest MenW outbreak occurred in Burkina Faso, in 2002, prior to the use of PsA-TT, MenW epidemics re-emerged in the African meningitis belt in 2010–2014 [40]. In 2016, a MenW outbreak occurred in Togo and northern Ghana [30]. 

While vaccination is the main strategy to control meningococcal outbreaks, vaccination may also change the patterns of outbreaks [6]. In order to monitor trends in disease and outbreaks, it is important for countries to have good surveillance systems in place [30,113].

## 5. Future Perspectives

Exciting perspectives for the future include the development of an affordable, multivalent vaccine for use in Africa to provide optimal protection against MenC, MenW, and MenX outbreaks. A MenACWYX conjugate vaccine is currently being developed and planned to be licensed in the coming years. The development of other multivalent combination vaccines, such as MenABCWY, is also ongoing. 

In light of the development of new or improved meningococcal vaccines, considerable research has been dedicated to new vaccine delivery systems. Nanoparticle systems are one example of a novel and promising technology for antigen delivery. In a MenA nanoparticulate vaccine formulation, the polysaccharide polymers are encapsulated in a biodegradable albumin-based material that slowly releases the antigens. The MenA nanoparticulate vaccine supplemented with MF59 or Alum adjuvant nanoparticles induced significantly higher surface expression of antigen presentation markers and co-stimulatory molecules in murine dendritic cells versus other adjuvant or non-adjuvanted nanoparticles [114]. An additional promising delivery system is the Generalized Modules for Membrane Antigens (GMMA), an OMV-based delivery system, whereby the GMMA serves as a carrier for heterologous polysaccharide or protein antigens. MenA and MenC polysaccharides conjugated to GMMA induced higher immune responses as compared with conventional conjugates in mice [115]. These new delivery systems may enhance immunogenicity, require fewer injections, and less stringent storage conditions, and come at a lower cost as compared with conventional vaccine formulations. Although further research is needed, these novel technologies are expected to contribute to and help to improve global immunization strategies in the future. 

Continued international efforts to control outbreaks are needed, such as the need for preparedness by having a vaccine registered and available for routine use in national immunization programs and stockpiled to be ready in case of outbreaks.

Further research is needed to evaluate the duration of protection and waning effectiveness after meningococcal conjugate and protein-based vaccines in order to determine the optimal timing of the booster dose.

Herd protection is a key factor in the high success of the MenC conjugate vaccine program in the UK and other countries. The impact of quadrivalent conjugate vaccines and protein-based vaccines on nasopharyngeal carriage and their ability to induce herd protection needs further evidence.

A visionary goal for the future is a world free of meningitis. To achieve this, the WHO and multiple partners launched a global strategy to defeat meningitis by 2030, and one of the key elements of this ambitious plan is to prevent bacterial meningitis through vaccination [116].

## 6. Conclusions

Despite the tremendous success in the control of meningococcal disease by vaccines, IMD still remains to be a major public health concern in many parts of the world. Vaccine introduction and coverage are variable. While some countries have implemented meningococcal vaccines via NIPs, some have only recommended vaccines to high-risk groups, travelers, or in response to outbreaks. Due to increased understanding, vaccine recommendations are continuously evolving, and newer vaccines are under development, with the aim to help achieve global meningitis control.

## Trademarks

*Menjugate*: *Menitorix*, *MenHibrix*, *Menveo*, and *Bexsero* are trademarks owned by or licensed to the GSK group of companies. *Nimenrix* is a trademark of the GSK group of companies, licensed to Pfizer. *NeisVac-C* and *Trumenba* are trademarks of Pfizer Inc. *Menactra, Menomune* and *MenQuadfi* are trademarks of Sanofi Pasteur. *Meningitec* is a trademark of Nuron Biotech. *MenAfriVac* and *NmCV-5* are trademarks of the Serum Institute of India Private Ltd. *VA-MENGOC*-BC is a trademark of the Finlay Institute, Cuba. *MenBvac* is a trademark of the Norwegian Institute of Public Health. *MeNZB* is a trademark of Novartis.
